# Wave Dispersion and Attenuation on Human Femur Tissue

**DOI:** 10.3390/s140815067

**Published:** 2014-08-15

**Authors:** Maria Strantza, Olivia Louis, Demosthenes Polyzos, Frans Boulpaep, Danny van Hemelrijck, Dimitrios G. Aggelis

**Affiliations:** 1 Department of Mechanics of Materials and Constructions, Vrije Universiteit Brussel, Pleinlaan 2, 1050 Brussels, Belgium; E-Mails: maria.strantza@vub.ac.be (M.S.); frans.boulpaep@vub.ac.be (F.B.); danny.van.hemelrijck@vub.ac.be (D.H.); 2 Department of Radiology, UZ Brussel,Vrije Universiteit Brussel, Avenue du Laerbeek 101, 1090 Brussels, Belgium; E-Mail: olivia.louis@uzbrussel.be; 3 Department of Mechanical Engineering and Aeronautics, University of Patras, Panepistimioupolis Rion, 26500 Patra, Greece; E-Mail: polyzos@mech.upatras.gr

**Keywords:** ultrasound, frequency, acoustic emission, waveform, dispersion, bone

## Abstract

Cortical bone is a highly heterogeneous material at the microscale and has one of the most complex structures among materials. Application of elastic wave techniques to this material is thus very challenging. In such media the initial excitation energy goes into the formation of elastic waves of different modes. Due to “dispersion”, these modes tend to separate according to the velocities of the frequency components. This work demonstrates elastic wave measurements on human femur specimens. The aim of the study is to measure parameters like wave velocity, dispersion and attenuation by using broadband acoustic emission sensors. First, four sensors were placed at small intervals on the surface of the bone to record the response after pencil lead break excitations. Next, the results were compared to measurements on a bulk steel block which does not exhibit heterogeneity at the same wave lengths. It can be concluded that the microstructure of the tissue imposes a dispersive behavior for frequencies below 1 MHz and care should be taken for interpretation of the signals. Of particular interest are waveform parameters like the duration, rise time and average frequency, since in the next stage of research the bone specimens will be fractured with concurrent monitoring of acoustic emission.

## 1. Introduction

Elastic wave measurements in heterogeneous media have always been a challenge. The reason is that apart from the mechanical and physical properties like elastic modulus, Poisson's ratio and density, wave parameters depend on additional material parameters and geometric characteristics. Media like cortical bone are of the most complex in this regard due to the porous microstructure, the varying properties (porosity, stiffness) from the outside (periosteum) to the inside (endosteum) and saturation with marrow. Apparently the thin and curved geometry induces additional plate wave dispersion. This study presents and discusses elastic wave measurements conducted in human femur bones. Multiple sensors were used to capture the transient response after the excitation at different positions and evaluate wave parameters like velocity, dispersion and attenuation. The aim is not to strictly correlate the wave characteristics with specific tissue parameters like thickness, age, healing, osteoporosis, possible tumor size or other deteriorating factors as is the scope of several studies [1–9]. A strong motivation for the research is to highlight the complexity of wave propagation in bone tissue. So far, this complexity limits the usefulness of the waveform to the first arrival signal in clinical studies. Therefore, all the information contained in the full waveform and the multiple modes is not exploited in full. In order to achieve this, the complexity should be shown and studied in order to gain background on the propagating modes and their connection with physical properties.

Additionally, this ultrasonic study is a preparation for the acoustic emission (AE) monitoring that will follow during fracture experiments on the same specimens. Femoral head fracture is a serious reason of mortality or loss of life quality for great numbers of elderly people. Since this is a subject of surgical repair, the way of fracture and the failure patterns are of great interest to the medical community. Characterization of mechanical and fracture properties (mechanical stiffness, load level for initiation of cracking and for final failure, fracture patterns, *etc.*) are important pieces of information for any engineering material, supplied by AE. For human bone this becomes more crucial, due to its importance for life and the fact that surgical repair of the fractured surfaces gains a lot from the understanding of fracture development and pattern even under controlled laboratory conditions in cadaveric specimens. Since AE studies will be conducted with the specific sensors on the femur specimens, it is imperative to make an ultrasonic study with the same setup. This is due to the strongly heterogeneous and dispersive nature of the material. The elastic pulse emitted by a cracking event is distorted throughout its propagation in such a heterogeneous medium. The medium complexity is due to the irregular porosity, the plate shape and the curvature [10]. In such media when a burst of energy goes into the formation of elastic waves, the different modes tend to separate due to the different velocities of the various frequency components. Failure to take this into account may lead to false characterization of the AE behavior in a material which is anyway relatively new to AE assessment. Indeed, although extensive AE studies have seen publicity concerning structural materials with satisfactory results concerning crack characterization (see indicatively [11,12]) the same does not hold for bone tissue where the AE waveform shape cannot a priori be connected to a fracture mode under a given load. Crucial parameters like the frequency or the duration of the AE waveforms which carry information on the cracking source will exhibit differences due to the propagation distance through the volume of the bone. This study concerns one sided ultrasonic measurements, capturing the wave propagating on the surface (axial transmission) and not through the thickness of the bone. A recent comprehensive review on the axial ultrasonic measurements of long cortical bone is given in [13]. So far only the first arrival signal has reached clinical studies. The time of flight has been related to thickness [10] as well as the strength of the femoral neck [14]. The stiffness matrix has been measured based on the pulse velocities *in vitro* [15]. Wave mode extraction and identification has been attempted in surface measurements [10]. Two main wave packets have been identified, one fast and one slow with strong dispersion having quite a large difference in their velocities [13,15–17]. Simulation studies have shown that the velocity of first arrival of the signal is expected to increase with frequency in the range between 500 kHz and 5 MHz [18], while the scattering in the microstructure has been analyzed based on effective medium approaches [6] as well as in the framework of enhanced elastic theories [19].

## 2. Experimental Section

This study was performed on five femur specimens excised from five cadavers. All the cadavers were obtained at the Anatomy Department of the School of Medicine of the Vrije Universiteit Brussel and had been preserved using vessels injections of formol solution. Age ranged from 73 to 95 years.

**Figure 1.**
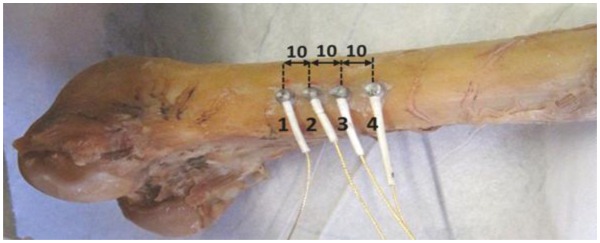
Acoustic emission sensors placed on the femur diaphysis (dimensions in mm).

The nature of the preliminary results (separating burst in the waveforms) imposed the use of multiple receivers in order to record the changes during propagation. The separation distance between the sensors was 10 mm (see Figure 1) and the pulse was excited by fractures of mechanical pencil leads of HB 0.5 approximately 5 mm in front of the first sensor. Excitation of elastic waves with pencil lead breaks is a common way either to check the performance of AE sensors [20] or to conduct ultrasonic studies [21]. The length and the hardness of the pencil lead are prescribed in order for the excitation to be repeatable. The edge of the lead is placed on the surface at the specified point and then it is broken by a gentle push. Care should be taken that the whole mechanical pencil does not touch the surface and creates extra noise signals. This is a realistic excitation, as it originates from the fracture of lead, and it carries quite a broad band of frequencies. Therefore, it can excite multiple modes, as opposed to narrow spectrum which is helpful for generating fewer modes or even a unique mode [22]. The sensors were of Pico type with a broadband response and maximum sensitivity at 450 kHz. The electric waveforms were pre-amplified by 40 dB and recorded in four synchronized channels with sampling rate of 10 MHz in the acquisition board (Mistras Holdings micro-II, 8 channels). Acoustic coupling was enhanced by Vaseline grease and the sensor array was placed on areas of the femur that were flat enough in order to avoid contact problems. The sensors were attached repeatedly in different spots to examine different wave paths. Measurements were conducted in five different femur specimens. Example of the sensor positioning is shown in Figure 1. The set up was first applied to a bulk steel specimen of thickness equal to 85 mm, in order to get a reference of a “homogeneous” medium with microstructure and thickness not interacting with the propagated wavelengths. Considering the frequencies of approximately 150 to 300 kHz excited by the lead break, the produced wavelengths are between 18 and 35 mm for longitudinal and 9–18 mm for Rayleigh. Therefore, there is no interaction with the thickness, something that excludes the possibility that the measured waves in the steel specimen are plate waves. On the contrary the thickness of the cortical bone is between 5 and 7 mm, strongly interacting with the developed wave lengths resulting in the propagation of plate waves in the specific experiment. It should be mentioned in any case that bulk waves in bone tissue can also be supported but for higher frequencies as has been demonstrated in a previous study [23], where the frequency of 1 MHz allowed the propagation of Rayleigh and longitudinal waves in bone with cortical bone thickness of 6.5 mm.

## 3. Results and Discussion

### 3.1. Waveforms

**Figure 2.**
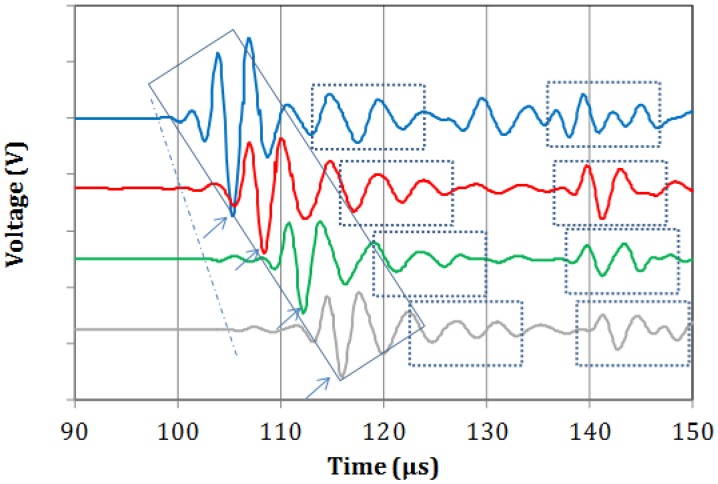
Elastic waveforms on the surface of bulk steel specimen.

Typical waveforms from the four sensors recorded on the metal specimen are shown in Figure 2. A weak onset is seen in all of the waveforms trailed by a much stronger portion of the waveform. These two bursts can presumably be identified as the longitudinal and Rayleigh waves respectively as the thickness of the steel block is too large to support plate waves in this frequency range. Since Rayleigh waves travel on a lower velocity, the 2nd bursts arrives at increasingly later times as the distance between excitation and receiver increases from sensor 1 to 4. Taking into account the distance between the sensors and the delay between the onset points (see dash dot line), the velocity of the longitudinal mode is calculated at 5464 m/s. This value is typical of steel material and it was repeatable on three different measurements on the material. It can be seen that the Rayleigh burst maintains a similar pattern throughout its propagation from the first to the last sensor (inside the diagonal rectangle). This is indicative of the homogeneity of the material and makes it identifiable throughout its propagation [24,25]. Based on a reference peak (*i.e.*, the strong negative indicated by arrows) the velocity of this mode is 2779 m/s. Considering the longitudinal velocity and the thickness of the metal block, reflections are expected at approximately 18–20 μs. The first and second reflections can be seen in the dashed rectangular boxes of Figure 2, with the first trailing or slightly overlapping with the Rayleigh burst.

**Figure 3.**
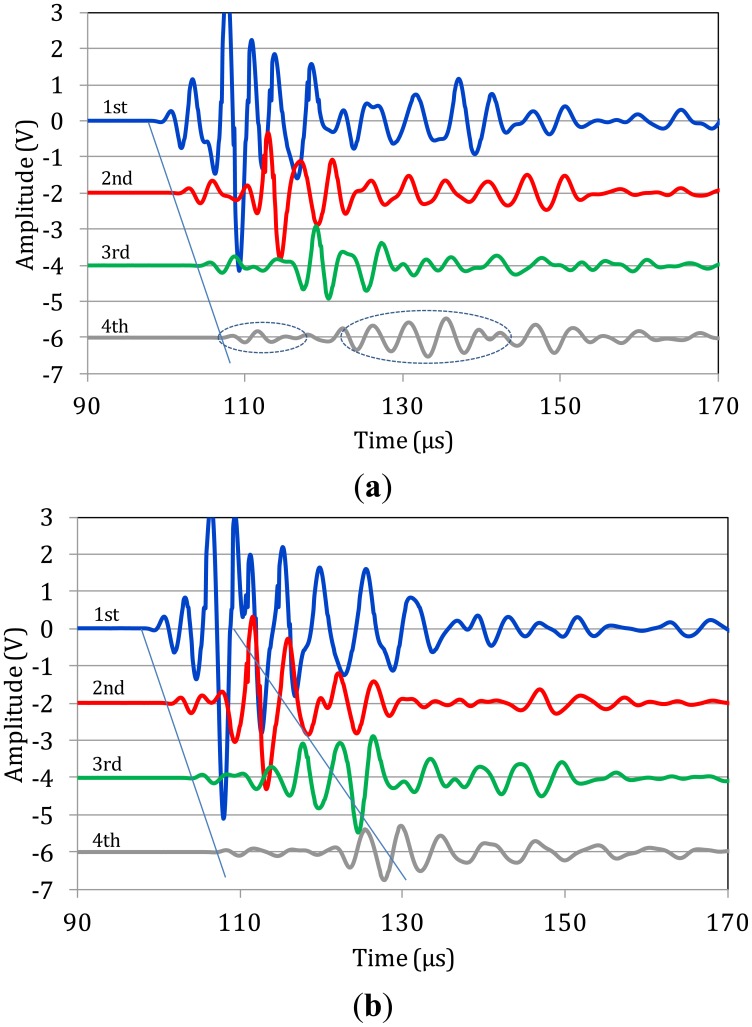

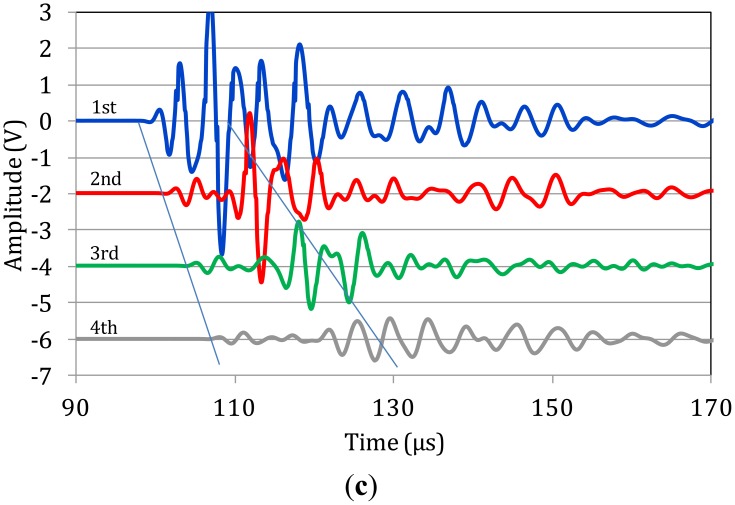
Elastic waveforms on the surface of three different femur bone specimens (**a**), (**b**) and (**c**).

Figure 3a–c shows corresponding typical measurements on bone specimens. Focusing first on Figure 3a one can see that as the propagation distance increases (from 1st to 4th sensor) a mode separation becomes evident. At the 4th sensor it is clear that a high-amplitude, low-frequency wave follows the initial weak arrivals (see ellipses). This separation is also seen on the waveforms of the 3rd and 2nd sensors, while on the 1st, the signals have not separated due to the short distance from the excitation. The exact same trend is seen for the next two cases of Figure 3b,c. The separation of different modes becomes more evident as the wave propagates further to the 4th receiver, while the duration of the large amplitude burst seems to stretch in time. Additionally, a “tail” is noted after the large amplitude mode, which can be the result of multiple reflections of bulk waves.

Focusing on the onset of the waveforms the velocity of the quick mode is measured between 3000 to 4000 m/s in all of the five different specimens and the individual ten measurements on them. This value is close to structural materials like mortar or concrete and is strongly related to the stiffness properties of the material. If one focuses on a reference point of the large amplitude burst (highest peak) the average value is approximately 1,500 m/s with a standard deviation of 12.5% of the average. These values coincide with measurements described in the literature on human bone specimens for the first two identified modes [10,16,17,26]. The major frequencies of the recorded waves are between 150 and 300 kHz, corresponding to wavelengths of 12 mm to 25 mm for a velocity of 3800 m/s of the fast mode and to 6 to 12 mm for the velocity of the slow mode. Since the thickness of the cortical bone is similar or smaller than the wave length (5 to 7 mm) these waves cannot be considered bulk waves as in the case of steel. They are reasonably assumed to be the first plate wave modes as discussed in a following section.

### 3.2. Amplitude Decay

**Figure 4.**
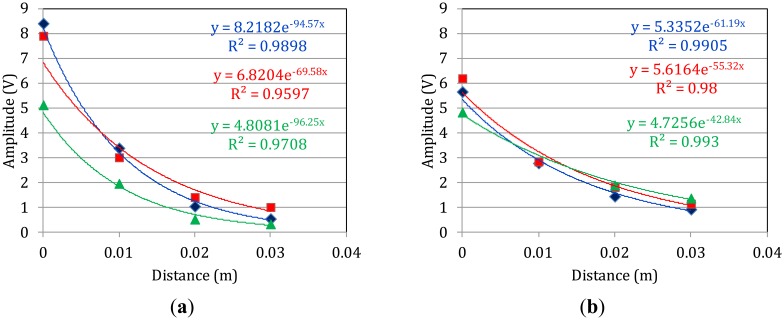
Amplitude *vs.* propagation distance curves for attenuation calculation. Specimens with average thickness of (**a**) 7.1 mm and (**b**) 5.8 mm.

Apart from wave velocity, another important parameter related to the stiffness and general structural condition of a material is attenuation or the rate of amplitude decay [27]. The well-established way to derive this parameter is by fitting an exponential decay line over the maximum amplitude *vs.* propagation distance plot. In the specific case, since the second burst of the waveform is the strongest, this procedure results in the decay rate of the specific burst. Figure 4 shows indicative cases from two specimens. As reasonable there is a substantial decay of the amplitude for each successive sensor (steps of 10 mm apart). Figure 4a,b, concern the femur samples with the highest thickness of 7.1 mm and the lowest thickness of 5.8 mm respectively. The thickness values stand for the average of five measurements throughout the circumference and were derived by the magnified photographs from optical microscope as will be discussed in Section 3.5. Each graph includes three independent measurements for each femur specimen. The fitting by exponential function results in quite strong correlation coefficients of more than 0.95 for almost all the cases. It is certain that even with the same specimen, different measurements result in different values due to the strong heterogeneity and local variability of the properties. However, it can still be noted that the specimen with the largest thickness exhibits the highest decay rate values (70 to 96 m^−1^, while the decay for the thin specimen ranges between 42 and 61 m^−1^. The rate of decay of specimens with intermediate thickness lies between these two ranges. This trend is related to the geometric spreading of the wave beam [25]. In thick specimens naturally the wave energy is distributed in larger volume. This decreases the amplitude contrary to the case of thin material where the wave energy is retained within limited material volume. It is worth to mention that the velocity of the two bursts follows the inverse trend with attenuation. Specifically for the sample with the low thickness of 5.8 mm, the velocities of the two modes are 3434 m/s and 1267 m/s. For the thick specimen of 7.1 mm, the corresponding velocities are 3137 m/s and 1037 m/s.

### 3.3. Dispersion and Attenuation Dependence on Frequency

**Figure 5.**
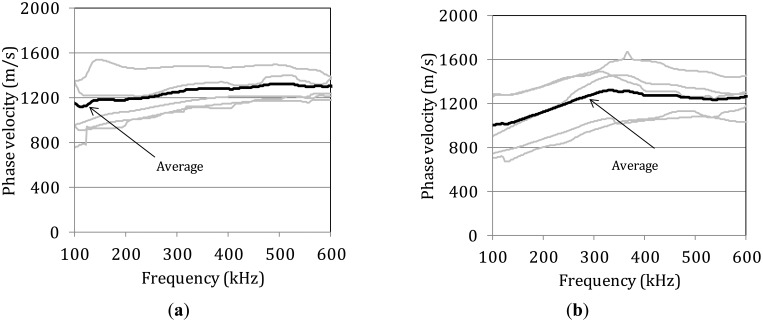
Dispersion curves from specimens with thickness (**a**) 7.1 mm and (**b**) 5.8 mm.

Based on the spectral content of the waveforms the phase velocity *vs.* frequency curve can be calculated [28]. Since it is obvious that different modes propagate, and in order to have reliable results the analysis was focused only on the large amplitude burst, forcing the rest of the waveform points to zero [29,30]. This procedure involves calculating the phase of the FFT of two waveforms (initial, *i.e.*, sensor 1 and final, *i.e.*, sensor 4), unwrapping of the phase information and calculation of the phase difference for each of the frequency components. This phase difference is proportional to the velocity of the frequency component and therefore, the dispersion curve is directly derived [28]. In Figure 5a several dispersion curves from different measurements on the femur specimen with thickness 7.1 mm are presented. The different curves exhibit strong experimental scatter which can be attributed to the heterogeneous structure of bone tissue. The heterogeneity makes any measurement unique even on the same specimen. In most of them a general increase of phase velocity is noted up to 500 or 600 kHz. The average of the lines is also drawn in the graph to more reliably show the increasing trend, without necessarily meaning that it is representative of the whole volume of the material. The average increase of wave velocity for this band of frequencies is more than 200 m/s from 1110 m/s to more than 1320 m/s. Figure 5b shows corresponding curves for the specimen with the lowest thickness of 5.8 mm. The scatter is similarly strong but there is a common trend of increase from 100 kHz to 400 kHz. In this case the average curve starts at less than 1000 m/s and increases up to 1320 for 350 kHz. The dispersive behavior of the bone specimens is in contrast with the metal dispersion curve which is a nearly straight line at the level of 3000 m/s [31]. This result is in good correspondence with recent measurements in porcine femur for the band of 150 kHz to 250 kHz that show a slight increase of group velocity for both modes which are again identified as the first symmetric and antisymmetric respectively [32]. The dependence of the phase velocity on the frequency shows the key role of the microstructure on wave propagation.

**Figure 6.**
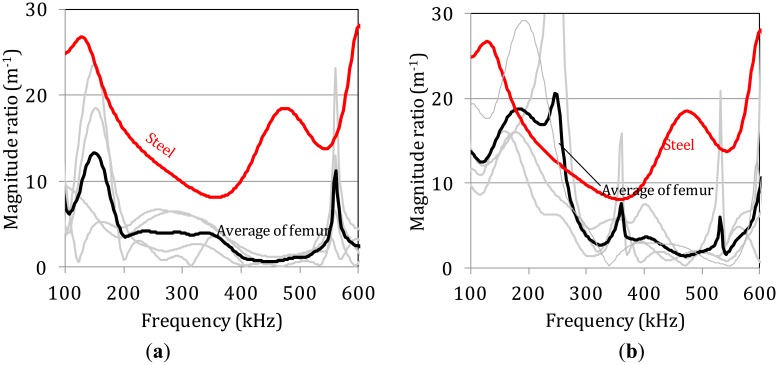
Magnitude decay *vs.* frequency curves derived from femur specimens with average thickness (**a**) 7.1 mm and (**b**) 5.8 mm.

Correspondingly, the attenuation behavior of the same specimens was assessed by comparing the frequency dependent magnitude of the FFT of the waveforms of the last receiver to the first, which acts as reference, and is depicted in Figure 6a and b. Specifically, the FFT of the waveform collected at the last receiver (A_4_) was divided to the FFT of the first (A_1_) and this was normalized to the distance X, between them (30 mm or 0.03 m) in order to express the magnitude ratio, a(f), over the propagation distance:

(1) $$a\left(f\right)=\frac{1}{X}\left(\frac{A_{4}}{A_{1}}\right)$$

The experimental scatter of the curves is even higher between the different bone specimens while the average curves clearly show a decrease of the magnitude for the bands higher than 250 kHz compared to below 250 kHz. The corresponding curve for steel is much smoother and higher due to the relatively homogeneous nature of the material. Despite the scatter, the average magnitude rate for thickness 7.1 mm (Figure 6a) is lower than for 5.8 mm (Figure 6b) for almost all the frequency bands and especially below 300 kHz. This trend is in accordance with the amplitude decay measured by the maximum amplitude of the signal in the previous section.

### 3.4. Wavelet Analysis

**Figure 7.**
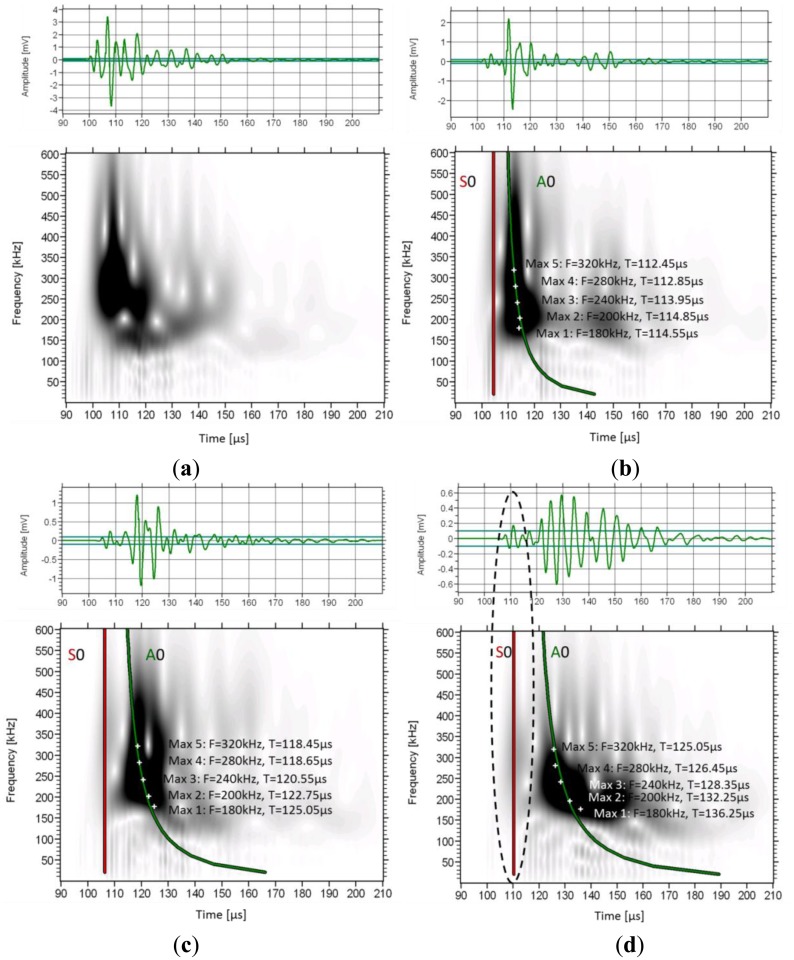
Waveforms and its wavelet transformation for (**a**) 1st sensor, (**b**) 2nd sensor, (**c**) 3rd sensor and (**d**) 4th sensor. The average thickness of cortical bone is 6.25 mm.

The frequency content in different parts of the waveforms can be visualized by means of wavelet analysis. It makes possible to identify the time window when a frequency band is dominant. The wavelet transform (WT) works by breaking the signal into shifted and scaled versions of the original wavelet (fast decaying mathematical function) [33]. To analyze any signal, several different original (“mother”) wavelets can be selected. Herein, the software used (AGU-Vallen Wavelet) employs the “Gabor” Wavelet, which has been used for analysis of elastic wave signals [34]. In Figure 7 the wavelet analysis of consecutive signals on a femur specimen from a single excitation is depicted as received by sensors 1 to 4.

The WT based on the 1st receiver reveals a concentrated intensity between 100 and 120 μs, with frequencies from 100 kHz to approximately 500 kHz. In this case no mode separation can be assumed. As the wave propagates to the next receivers, the higher frequency components start to wear down and the intensity spreads to later time windows. In sensor 4 (Figure 7d), although the higher intensity is seen at the location of the second low frequency burst (120 to 160 μs), some energy is still visible earlier corresponding to the higher frequency, lower amplitude initial part of the signal which arrives at approximately 110 μs (see the connection to the waveform with the dash ellipse). On the same graphs the maximum intensity for certain frequencies is also seen by white cross symbols. It is clear that as the wave propagates further from sensor 1 to 4, the maxima of the lower frequencies shift to later times compared to the higher frequency components. As an example, the peak at 180 kHz arrives 2.1 μs later than the peak of 320 kHz in sensor 2 (Figure 7b), while in sensor 4 (Figure 7d), the delay between them is more than 10 μs. This increasing delay between different frequency components indicates the strong dispersion due to the microstructure, with higher frequencies propagating on higher speed.

As an approach to theoretically explain the observed results, simulated dispersion curves are also added on the graphs. These concern the first symmetric and antisymmetric Lamb modes and were constructed based on material with longitudinal wave velocity of 3800 m/s and shear velocity of 1800 m/s. These values were selected to approximately match the pulse velocities measured by the onset and the maximum peak as already mentioned and they coincide with typical values used in literature [13,15,17]. The thickness was considered 0.3 mm and no curvature was assumed (simple plate geometry). The first (red line) corresponds to the 1st symmetric mode (S0) and the green to the antisymmetric (A0). As the distance of propagation increases, the lines get further apart due to the different velocities of the modes. One noticeable detail is that the line of the A0 coincides with the maxima of the frequencies of the strong 2nd burst. This is a strong indication that this 2nd burst is actually the 1st antisymmetric mode since the arrival times of its maxima fall on the predicted lines. However, it must be stressed, that the Lamb dispersion equations assume constant thickness and therefore, cannot take into consideration the actual irregularities of the bone structure and its curvature.

**Figure 8.**
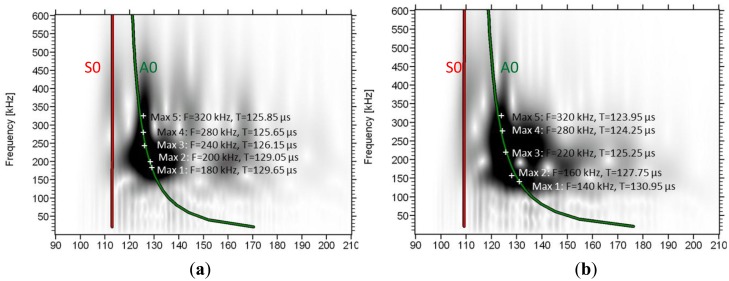
wavelet transformation for the 4th sensor for specimens with average cortical thickness (**a**) 5.45 mm and, (**b**) 6.65 mm.

The trend is similar for most of the bone specimens. Two other examples of wavelet transformation are shown below (Figure 8) from specimens with average thickness of 5.45 mm and 6.65 mm. In these cases as well, the maxima of the frequency content as revealed by the wavelet analysis are close to the theoretical dispersion curve calculated with a thickness much less than the cortical thickness of the bone (0.3 mm) as will be seen in the next section.

### 3.5. Thickness Considerations

An important point for discussion is that the assumed thickness for calculation of the dispersion curves was much less than the physical thickness of the bone (0.3 mm while the thickness measured on the microscope is over 4 mm). Values around 3 to 4 mm have been used in other cases for simulation [10,17,26]. In this case the theoretical dispersion curves agree with the experimental results for a much smaller thickness (approximately one tenth of the actual). Though this needs further elaboration, it should be kept in mind that the porosity of femur strongly increases from the external surface inwards. Especially for aged specimens (older than 80 years of age, as is the case for this study) the porosity starts typically at 5% and increases to more than 25% in the inner surface. Additionally, the stiffness decreases dramatically from the periosteum (external layer) to endosteum (internal layer) especially for the older group (above 80 years) by about 30% [35]. Furthermore, in “athletic” species like human, the external layer (periosteum) of the femur is much stronger relatively to non-athletic species. Also due to the *in vitro* nature of the study the specimens cannot be considered moisture saturated as would be while inside a living body. Saturation would enable propagation in deeper layers of the material certainly altering the wave patterns [17,19,36]. All these parameters indicate that in the present case the external layer of the femur is much stiffer and bears most of the energy of wave propagation showing that possibly the thickness used for theoretical dispersion curves should not be the actual full thickness of the bone but a fraction of it. Using the full thickness of 5 to 7 mm as measured by micro-photographs resulted in theoretical dispersion curves with no connection to the arrivals of the frequency components of the waveform.

**Figure 9.**
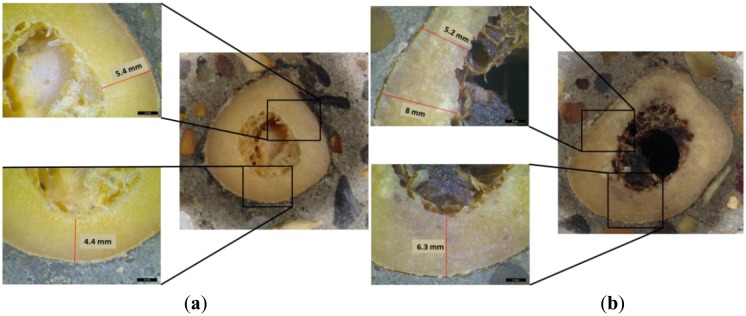
Cross section of a femur with (**a**) small thickness and with (**b**) large thickness.

A femur cross-section consists of three layers: the periosteum or outside skin of the bone, the hard compact bone (cortical) and the bone marrow. Feik *et al.* demonstrated that cross-sections of cortical bone clearly show age-related differences in the thickness [37]. The compact bone area is highest in young ages and lower in older ones [38]. As it has already been discussed previously, the age of the cadavers ranged from 73 to 95 years. This means that the cortical bone area is expected to be relatively low. Cross sections of two femur specimens of the current study can be seen in Figure 9a and b. The thickness measurement is based on the cortical layer. The cortical thickness is derived by the magnified photographs from optical microscope. Those two femur specimens were selected based on the size differences. They correspond to the specimens with the largest thickness (7.1 mm in average) and the lowest at 5.8 mm. Those values have been measured as average of five measurements at the cross-section surface (see indicative values in Figure 9). In any case, it is clear from the pictures that the thickness in both femurs is not uniform. This is due to the inhomogeneous geometry of the femur tissue.

### 3.6. Other Waveform Parameters

**Figure 10.**
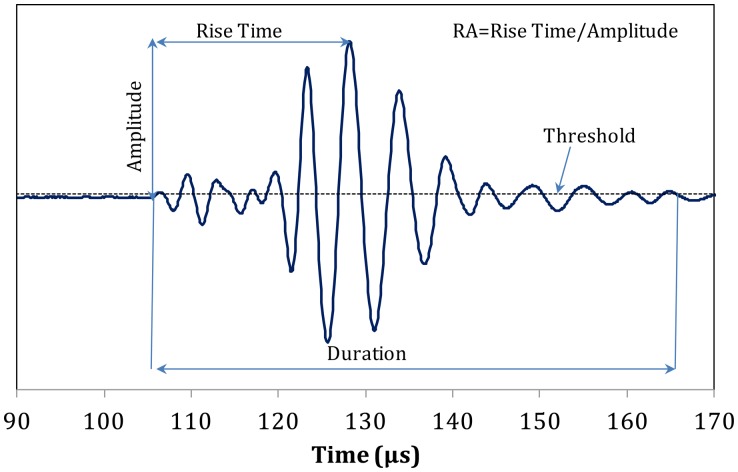
Typical AE waveform with basic parameters.

Apart from wave velocity and attenuation, other waveform parameters are also important mainly for AE monitoring. Some of them are the RA value and the average frequency (AF). RA is the ratio of Rise Time over the maximum amplitude (see Figure 10) and AF is the number of threshold crossings over the duration of the signal. Additionally, central frequency (CF) is the frequency with the maximum magnitude in the frequency domain. In engineering materials a shift to high RA values and low frequency indicates a shift from tensile to shear cracking sources [39].

**Figure 11.**
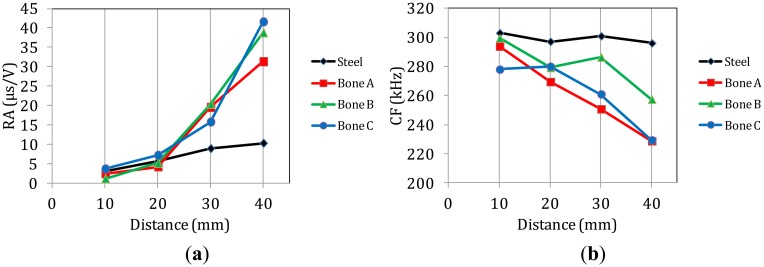

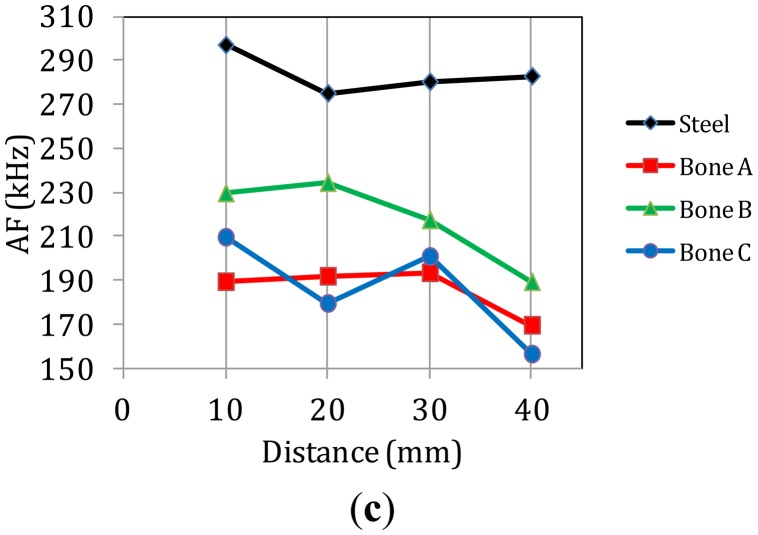
(**a**) RA, (**b**) CF and (**c**) AF of waveforms at different sensors on femur and steel.

Figure 11a shows the RA for the successive sensors for steel and different bone specimens as a response to the simulated fracture of the pencil lead. The RA of the waveforms captured on steel increases smoothly mainly due to the slower propagation of the Rayleigh that increases the delay between the onset and the maximum (see waveforms of Figure 2). The corresponding increase for the bone specimens is much stronger. The values at the last receiver are more than 10 times higher than the first, a change that occurs for additional propagation of just 30 mm. Specifically, nearby the excitation, RA is always less than 5 μs/V while 30 mm further it approaches 45 μs/V. This alteration by a factor of 10 is too strong and would certainly mask any change owing to the actual fracture mode. Concerning the central frequency (Figure 11b), the pulses in steel do not seem to systematically lose their content with distance since all of them are around 300 kHz. However, again the femur material has a much stronger influence on the frequency content as the CF starts between 280 kHz and 300 kHz for the transducer near-by the excitation but decreases to around 250 kHz 30 mm away for most of the measurements. Figure 11c shows the corresponding trend of AF which although is still a frequency indicator, it is measured through the threshold crossings of the time domain waveform. Despite its seemingly rough and threshold-dependent nature it has proven very powerful for the characterization of cracking modes in different materials. Again a strong decrease is depicted for the bone specimens while the pulses in steel do not seem to clearly lose their AF. These changes in waveform parameters for additional propagation of just a few mm highlight the importance of waveform distortion in AE analysis in heterogeneous media like bone. The need to take distortion into account has been demonstrated for engineering materials where the waveform parameters are used for classification of fracture modes and any distortion of the shape due to scattering is crucial [40]. However, it has not been considered in bio-materials although they are more complex. Therefore, if the aim of AE monitoring is not limited to measuring the accumulated number of AE activities and more specific information is sought for the dominant fracture mechanisms of the tissue and how they are triggered relatively to the applied load, the effect of distortion should be cleared or at least taken into consideration [41].

## 4. Conclusions

This study discusses elastic wave propagation in human femur bone specimens. Measurements are conducted with broadband acoustic emission transducers and the excitation is driven by pencil lead breaks. Strong dispersive and attenuative trends are observed with high frequencies propagating faster for the band up to about 500 kHz. This is attributed to the effect of microstructure and of the curved plate geometry as revealed by comparison with waveforms from a bulk and homogeneous metal block. Theoretical investigation concerning the identification of the specific wave modes is also conducted and compared with the energy intensity revealed by wavelet transformation of the experimental waveforms. The agreement is quite good if the theoretical calculations are conducted for a thickness equal to one tenth or less of the macroscopical thickness measured by microscope. This is not unreasonable considering the complex and varying nature of the mechanical and physical properties through the thickness of the bone. Microstructure has also a strong effect on the shape of the waveforms. This is crucial in medical applications where the highest possible degree of reliability and accuracy is necessary [42]. As the propagation distance increases, parameters like the rise time, central and average frequency exhibit strong changes. This stresses out that care should be taken for the interpretation of AE signals during fracture since the effect of distortion is accumulating and masks the original shape of the waveforms.

## References

[1] Sarvazyan A., Tatarinov A., Egorov V., Airapetian S., Kurtenok V., Gatt C.J. (2009). Application of the dual-frequency ultrasonometer for osteoporosis detection. Ultrasonics.

[2] Castellazzi G., De Marchi L., Krysl P., Marzani A., Tribikram Kundu T. Quantitative simulation of wave propagation in a human leg to support the ultrasonic non-invasive assessment of human bones.

[3] Bossy E., Talmant M., Laugier P. (2002). Effect of bone cortical thickness on velocity measurements using ultrasonic axial transmission: A 2d simulation study. J. Acoust. Soc. Am..

[4] Vavva M.G., Protopappas V.C., Gergidis L.N., Charalambopoulos A., Fotiadis D.I., Polyzos D. (2009). Velocity dispersion of guided waves propagating in a free gradient elastic plate: Application to cortical bone. J. Acoust. Soc. Am..

[5] Moilanen P. (2008). Ultrasonic guided waves in bone. IEEE Trans. Ultrason. Ferroelectr. Freq. Control.

[6] Potsika V.T., Grivas K.N., Protopappas V.C., Vavva M.G., Raum K., Rohrbach D., Polyzos D., Fotiadis D.I. (2014). Application of an effective medium theory for modeling ultrasound wave propagation in healing long bones. Ultrasonics.

[7] Nairus J., Ahmadi S., Baker S., Baran D. (2000). Quantitative Ultrasound: An Indicator of Osteoporosis in Perimenopausal Women. J. Clin. Densitom..

[8] Zheng R., Le L.H., Sacchi M.D., Ta D. (2007). Spectral ratio method to estimate broadband ultrasound attenuation of cortical bones *in vitro* using multiple reflections. Phys. Med. Biol..

[9] Giangregorio F. (2011). Contrast-Enhanced Ultrasound (CEUS) for Echographic Detection of Hepato Cellular Carcinoma in Cirrhotic Patients Previously Treated with Multiple Techniques: Comparison of Conventional US, Spiral CT and 3-Dimensional CEUS with Navigator Technique (3DNav CEUS). Cancers.

[10] Laugier P. Recent Advances in QUS Assessment of Bone Old Dreams—New Hopes.

[11] Shahidan S., Pulin R., Muhamad Bunnori N., Holford K.M. (2013). Damage classification in reinforced concrete beam by acoustic emission signal analysis. Constr. Build. Mater..

[12] Farhidzadeh A., Dehghan-Niri E., Salamone S., Luna B., Whittaker A. (2013). Monitoring Crack Propagation in Reinforced Concrete Shear Walls by Acoustic Emission. J. Struct. Eng..

[13] Määttä M. (2012). Assessment of Osteoporosis and Fracture Risk, Axial Transmission Ultrasound and Lifestyle-Related Risk Factors. PhD Thesis.

[14] Grimal Q., Grondin J., Guerard S., Barkmann R., Engelke K., Gluer C.-C., Laugier P. (2013). Quantitative Ultrasound of Cortical Bone in the Femoral Neck Predicts Femur Strength: Results of a Pilot Study. J. Bone Miner. Res..

[15] Pithioux M., Lasaygues P., Chabrand P. (2002). An alternative ultrasonic method for measuring the elastic properties of cortical bone. J. Biomech.

[16] Tran T.N.H.T., Stieglitz L., Gu Y.J., Le L.H. (2013). Analysis of Ultrasonic Waves Propagating in a Bone Plate over a Water Half-Space with and without Overlying Soft Tissue. Ultrasound Med. Biol..

[17] Ta D., Huang K., Wang W., Wang Y., Le L.H. (2006). Identification and analysis of multimode guided waves in tibia cortical bone. Ultrasonics.

[18] Nguyen V-H., Naili S. (2013). Ultrasonic wave propagation in viscoelastic cortical bone plate coupled with fluids: A spectral finite element study. Comput. Methods Biomech. Biomed. Eng..

[19] Protopappas V.C., Kourtis I.C., Kourtis L.C., Malizos K.N., Massalas C.V., Fotiadis D.I. (2007). Three dimensional finite element modeling of guided ultrasound wave propagation in intact and healing long bones. J. Acoust. Soc. Am..

[20] Ohtsu M. (2010). Recommendation of RILEM TC 212-ACD: Acoustic emission and related NDE techniques for crack detection and damage evaluation in concrete: 1. Measurement method for acoustic emission signals in concrete. Mater. Struct..

[21] Aggelis D.G., Shiotani T. (2007). Experimental study of surface wave propagation in strongly heterogeneous media. J. Acoust. Soc. Am..

[22] Luo W., Rose J.L. (2004). Lamb wave thickness measurement potential with angle beam and normal beam excitation. Mater. Eval..

[23] Le L.H., Gu Y.J., Li Y., Zhang C. (2010). Probing long bones with ultrasonic body waves. Appl. Phys. Lett..

[24] Aggelis D.G., Shiotani T. (2008). Surface wave dispersion in cement-based media: Inclusion size effect. NDT&E Int..

[25] Owino J.O., Jacobs L.J. (1999). Attenuation measurements in cement based materials using laser ultrasonics. J. Eng. Mech..

[26] Ta D., Wang W., Wang Y., Le L.H., Zhou Y. (2009). Measurement of the dispersion and attenuation of cylindrical ultrasonic guided waves in long bone. Ultrasound Med. Biol..

[27] Ranz J., Aparicio S., Romero H., Casati M.J., Molero M., González M. (2014). Monitoring of Freeze-Thaw Cycles in Concrete Using Embedded Sensors and Ultrasonic Imaging. Sensors.

[28] Sachse W., Pao Y.-H. (1978). On the determination of phase and group velocities of dispersive waves in solids. J. Appl. Phys..

[29] Dokun O.D., Jacobs L.J., Haj-Ali R.M. (2000). Ultrasonic monitoring of material degradation in FRP composites. J. Eng. Mech..

[30] Philippidis T.P., Aggelis D.G. (2005). Experimental study of wave dispersion and attenuation in concrete. Ultrasonics.

[31] Strantza M., Louis O., Polyzos D., Boulpaep F., Van Hemelrijck D., Aggelis D.G. Measurement of elastic wave dispersion on human femur tissue.

[32] Chen J., Su Z. (2014). On ultrasound waves guided by bones with coupled soft tissues: A mechanism study and *in vitro* calibration. Ultrasonics.

[33] Misiti M., Misiti Y., Oppenheim G., Poggi J. (2009). Matlab Wavelet Toolbox User's Guide.

[34] Hamstad M.A. An Illustrated Overview of the Use and Value of a Wavelet Transformation to Acoustic Emission Technology.

[35] Baron C. Ultrasonic guided waves in cortical bone modeled as a functionally graded anisotropic tube.

[36] Baron C., Naili S. (2010). Propagation of elastic waves in a fluid-loaded anisotropic functionally graded waveguide: Application to ultrasound characterization. J. Acoust. Soc. Am..

[37] Feik S.A., Thomas C.D.L., Clement J.G. (2002). Age-related changes in cortical porosity of the midshaft of the human femur. J. Anat..

[38] Stein M.S., Thomas C.D.L., Feik S.A., Wark J.D., Clement J.G. (1998). Bone size and mechanics at the femoral diaphysis across age and sex. J. Biomech..

[39] Ohtsu M. (2010). Recommendations of RILEM Technical Committee 212-ACD: Acoustic emission and related NDE techniques for crack detection and damage evaluation in concrete: 3. Test method for classification of active cracks in concrete structures by acoustic emission. Mater. Struct..

[40] Aggelis D.G., Shiotani T., Papacharalampopoulos A., Polyzos D. (2012). The influence of propagation path on acoustic emission monitoring of concrete. Struct. Health Monit..

[41] Aggelis D.G., Paschos N.K., Barkoula N.M., Paipetis A.S., Matikas T.E., Georgoulis A.D. (2011). Rupture of anterior cruciate ligament monitored by acoustic emission. J. Acoust. Soc. Am..

[42] Almeida M.S.D., Maciel C.D., Pereira J.C. (2007). Proposal for an Ultrasonic Tool to Monitor the Osseointegration of Dental Implants. Sensors.

